# *Hibiscus manihot* L. flower extract induces anticancer activity through modulation of apoptosis and autophagy in A549 cells

**DOI:** 10.1038/s41598-024-58439-3

**Published:** 2024-04-06

**Authors:** Minglu Xu, Mengxia Zhao, Miaomiao Zhu, Hongmei Yuan, Zhongzheng Li, Peishuo Yan, Chi Ma, Huabin Zhao, Shenghui Wang, Ruyan Wan, Lan Wang, Guoying Yu

**Affiliations:** 1 School of Chemistry and Chemical Engineering, Henan Institute of Science and Technology, Xinxiang, 453-003 Henan China; 2 State Key Laboratory of Cell Differentiation and Regulation, Henan International Joint Laboratory of Pulmonary Fibrosis, Henan Center for Outstanding Overseas Scientists of Organ Fibrosis, Institute of Biomedical Science, College of Life Science, Henan Normal university, 46 Jianshe Road, Xinxiang, 453007 Henan China

**Keywords:** *Hibiscus Manihot L* flower, ROS, A549 cell, Apoptosis, Mitophagy, Cancer, Cell biology, Drug discovery

## Abstract

Lung cancer is a major public health issue and heavy burden in China and worldwide due to its high incidence and mortality without effective treatment. It’s imperative to develop new treatments to overcome drug resistance. Natural products from food source, given their wide-ranging and long-term benefits, have been increasingly used in tumor prevention and treatment. This study revealed that *Hibiscus manihot* L. flower extract (HML) suppressed the proliferation and migration of A549 cells in a dose and time dependent manner and disrupting cell cycle progression. HML markedly enhanced the accumulation of ROS, stimulated the dissipation of mitochondrial membrane potential (MMP) and that facilitated mitophagy through the loss of mitochondrial function. In addition, HML induced apoptosis by activation of the PTEN-P53 pathway and inhibition of ATG5/7-dependent autophagy induced by PINK1-mediated mitophagy in A549 cells. Moreover, HML exert anticancer effects together with 5-FU through synergistic effect. Taken together, HML may serve as a potential tumor prevention and adjuvant treatment for its functional attributes.

## Introduction

Lung cancer is the second most common cancer in the world and the leading cause of cancer death, accounting for exactly 25% of all cancer deaths. According to GLOBOCAN 2022, more than 350 people die each day from lung cancer^1^. Despite some advances in the treatment of lung cancer in recent years, the 5-year survival rate for this disease remains less than 17%^2^. The complexity and heterogeneity of lung cancer leads to limited treatment strategies.

Traditional Chinese medicine has gradually taken into anticancer agents; for example, Lotus leaf flavonoids can induce apoptosis in A549 cells^3^, and dihydroisotanshinone can induce ferroptosis and apoptosis in lung cancer cells^4^. In addition, apigenin, as a dietary flavonoid, inhibits HepG2 cell growth and induces apoptosis of HepG2 cells in a time—and dose-dependent manner^5^. *Curcumae rhizoma* is a traditional herbal medicine, and research has found that the chemical components isolated from turmeric have anti-liver cancer effects^6^. *Hibiscus manihot* L. is an annual edible herb of the genus Okra in the malvaceous family. Pharmacological studies suggest that HML has multiple biological properties, including anti-inflammatory, antioxidant, antilipogenic and neuroprotective effects^7^. Flavonoids are the main biologically active components in HML flowers^8^. The HML aqueous extract contains polyphenols and phenolic acids, in addition to flavonoids, which was confirmed by HPLC–UV/DAD analysis^9^. Accumulating evidence suggests that polyphenols in plants have beneficial effects in the treatment and prevention of cancer^10,11^. In this study, we hypothesize that HML exert significant anticancer effects. HML flowers were extracted and determined the role and its mechanism of HML regulation of proliferation, migration, apoptosis and autophagy of lung cancer cells.

## Materials and methods

### Preparation extract of HML

The *Hibiscus manihot L.* flowers were collected from the farm of Xinxiang, Henan, China in May, 2019. The harvested plant material was morphologically identified at Henan Institute of Science and Technology by Professor Li Meng. The flowers collection followed relevant local, national, and international guidelines, permissions, or legislation and we obtained the necessary permissions from the governorate of Xinxiang. Flower samples were stored in a dry warehouse at room temperature, the dried flowers were powdered and extracted thrice with 90%, 95% and 99.9% edible ethanol for 24 h in a 50 °C water bath. The three extracts were pooled, concentrated and lyophilizate under vacuum at – 60 °C. Dissolved in PBS and filtered through a 0.22 μm membrane to prepare a 100mg/mL solution at – 20 °C for subsequent experiments.

### Cell culture

A549 cells (human lung adenovirus cell line) were purchased from the American Type Culture Collection (Shanghai, China), and BEAS-2B was purchased from Pricella (CL-0496). A549 cells were maintained in DME/F-12 medium, while BEAS-2B were cultured in DMEM –HIGH medium, supplied with 10% (v/v) fetal bovine serum (FBS), 1% streptomycin–penicillin in 5% CO_2_ at 37°C. Commercial standard of 5-FU(5-fluorouracil, HY-90006) at 100 μM as positive control.

### Cell viability assays

A549 and BEAS-2B cells were seeded in 96-well plates (5 × 10^3^ cells/well) and treated with various concentrations of HML for 48 h. Cell viability was tested using Cell Counting Kit-8. Cell viability and inhibition rate were calculated. Prism8 software was used to analyze the data.

### Colony formation assay

A549 cells were seeded in 6-well plates (2 × 10^4^ cells/well), plates and cultured 5% CO2 incubator at 37 °C for 24 h. After the incubation periods, the cells were treated with 400 μg/mL HML or PBS for 4 days, 7 days and 14 days, respectively. Colonies are fixed with glutaraldehyde (6.0% v/v), stained with crystal violet (0.5% w/v) and counted using a stereomicroscope.

### Wound healing assay

A549 cells were seeded in sterile 6-well plates (1.2 × 10^6^ cells/well) and grown overnight in the incubator. Cells were treated with HML (400 μg/mL). When the cells have grown to 90–100% confluence in the monolayer, draw a straight line on the cell surface with a sterile pipette tip. Wounds were photographed under an inverted research microscope (Leica D-35578, Wetzlar, Germany) at 0, 24, 48, and 72 h after injury. Image J was used to analyze the scratch area.

### EdU detection

A549 cells (2 × 10^4^ cells/well) were seeded into 96-well plates incubated with HML (400 μg/mL) in a 37 °C, 5% CO_2_ incubator. Cell proliferation was evaluated using the EdU Assay Kit (Ribobio). Positive cells were evaluated under a fluorescence microscope (Nikon, Japan).

### Transwell invasion assay

Cell transmigration assay was performed using a Transwell instrument (Corning). After treatment with HML (0, 400 μg/mL) for 24 h, then transfer the cells into Transwell. Cells (1 × 10^4^) in 100 μL of FBS-free medium were added to the upper chamber, while 600 μL of 10% FBS was added to the lower chamber. After 24 h of incubation, cells on the upper surface of the filter were removed. Filters were fixed with 4% paraformaldehyde and stained with crystal violet (0.5%). Three fields of view were then selected and counted under a light microscope.

### Senescence-associated beta-galactosidase staining

A549 cells (2 × 10^4^ cells/well) cultured in 24-well plates at 37 °C and 5% CO_2_ treated with 400 μg/mL HML or not. After 5 days, cells were washed with PBS and fixed in β-Gal fixative for 20 min. The fixed cells were maintained overnight at 37 °C (without CO_2_) with SA-β-Gal staining solution. Finally, green blue–colored cells were counted as a percentage of the total cell number and displayed as a percentage of cell senescence.

### Cell cycle analysis

Cells (2 × 10^5^/well) were seeded in 6-well plates. The cells were starved for 24 h at 37 °C, and then treated with HML for an additional 48 h. Cell cycle analysis was performed by flow cytometry (Becton Dickinson FACSCalibur, San Jose, CA). The DNA content in the G1, S, and G2/M phases was analyzed using Flowjo softwore.

### TUNEL staining

Cells (2 × 10^4^ cells/well) were seeded into 96-well plates, after treatment with HML (400 μg/mL) for 48 h, apoptosis was measured by terminal deoxynucleotidyl transferase dUTP nick end-labeling (TUNEL) assay using the DeadEnd™ fluorometric TUNEL system, according to the manufacturer’s instruction (40306ES60, YESEN). Images were visualized using an Axio Imager D2.

### Detection of ROS

The intracellular ROS level was assessed using a ROS assay kit (Applygen Technologies, Inc., Beijing, China). Briefly, the cells (HML at a concentration of 400 μg/mL for 48 h) were incubated with 10 μM DCFH-DA at 37 °C for 30 min in the dark. Following incubation, the cells were washed twice with PBS and the DCF fluorescence intensity was detected by flow cytometry (Becton Dickinson FACSCalibur, San Jose, CA) or fluorescence multiplanar reader (BioTek Synergy LX).

### Mitochondrial transmembrane potential (Δψm) assay

Cells was seeded in 6-well plates and treated with HML (400 μg/mL) for 48 h, then stained with JC-10 following the manufacturer's instructions (Solarbio, Beijing, China).The fluorescence intensity of JC-10 was then measured in a BioTek Synergy LX or flow cytometry. MMP was assessed using JC-1 (Dojindo Molecular Technologies, Tokyo, Japan) staining according to the manufacturer’s protocols and previous reports^12^.

### ATP luminescent cell viability assay

A549 was seeded in 96-well plates (5 × 10^3^ cells/well) and treated with HML (400 μg/mL), 5 FU (100 μM), and a combination of the two for 48 h, followed by medium removal. Then, 100 μL of normal medium was added again, and then 100 μL Working solution (CK18) was added. The mixture was stirred and mixed by BioTek Synergy LX, and incubated for 10 min at room temperature in the dark. The luminescence value was detected.

### Quantitative RT-PCR analyses

A549 cells were seeded in 6-well plates and incubated overnight at 37 °C. A549 cells were inoculated in 6-well plates, exposed to 0μg/mL or 400μg/mL HML for 24 h, and incubated at 37 ℃. TRIzol (Takara) was used to extract total RNA, and cDNA was transcribed using the GoScript™ Reverse Transcription System (Promega, A5001). The reaction was performed by using Quanti Nova SYBR Green PCR Kit (QIAGEN, 208052) on a Light Cycler 480 system (Roche). Relative mRNA levels were quantified by calculating the comparative 2-ΔΔCt method.

### Western blot analysis

A549 was seeded in 6-well plates (1.2 × 10^4^ cells/well) and treated with HML (0 μg/mL or 400 μg/mL) for 48 h. Total proteins were extracted from cells using RIPA buffer containing protease inhibitors. The protein concentration was evaluated with the Pierce BCA method. Then, each protein sample was separated by 8–12% SDS-PAGE, and the proteins were transferred to nitrocellulose membranes. After blocking in 5% skim milk, the membranes were probed with the following primary antibodies overnight at 4 °C. The membranes were then incubated with a horseradish peroxidase-conjugated secondary antibody for 1 h at room temperature. The blots were visualized by Odyssey. Software Version 5.2, LI-COR Biosciences.

### Immunofluorescence staining

After transfection, cells were treated with HML (0 μg/mL or 400 μg/mL) for 24 h and chloroquine (10 µM) for 6 h, and washed by PBS three times, then fixed with 4% paraformaldehyde for 15 min. The fixed cells were permeabilized with 0.3% penetration buffer for 5min. Later the cells were blocked with 10% goat serum at room temperature for 15 min. Nucleuses’ staining was carried out with DAPI for 5 min. The cells were photographed under a fluorescence microscope.

### Statistical analysis

All experiments were performed at least three times independently. The data were presented as means ± SEM. The differences between treatments were analyzed by one-way analysis of variance, followed by Dunnett’s multiple comparison method using Prism 8.0 (GraphPad Software, San Diego, CA, USA), and p < 0.05, p < 0.01, and p < 0.001 were used to indicate statistical significance.

## Results

### HML inhibited A549 cell growth and migration

**Figure 1:**
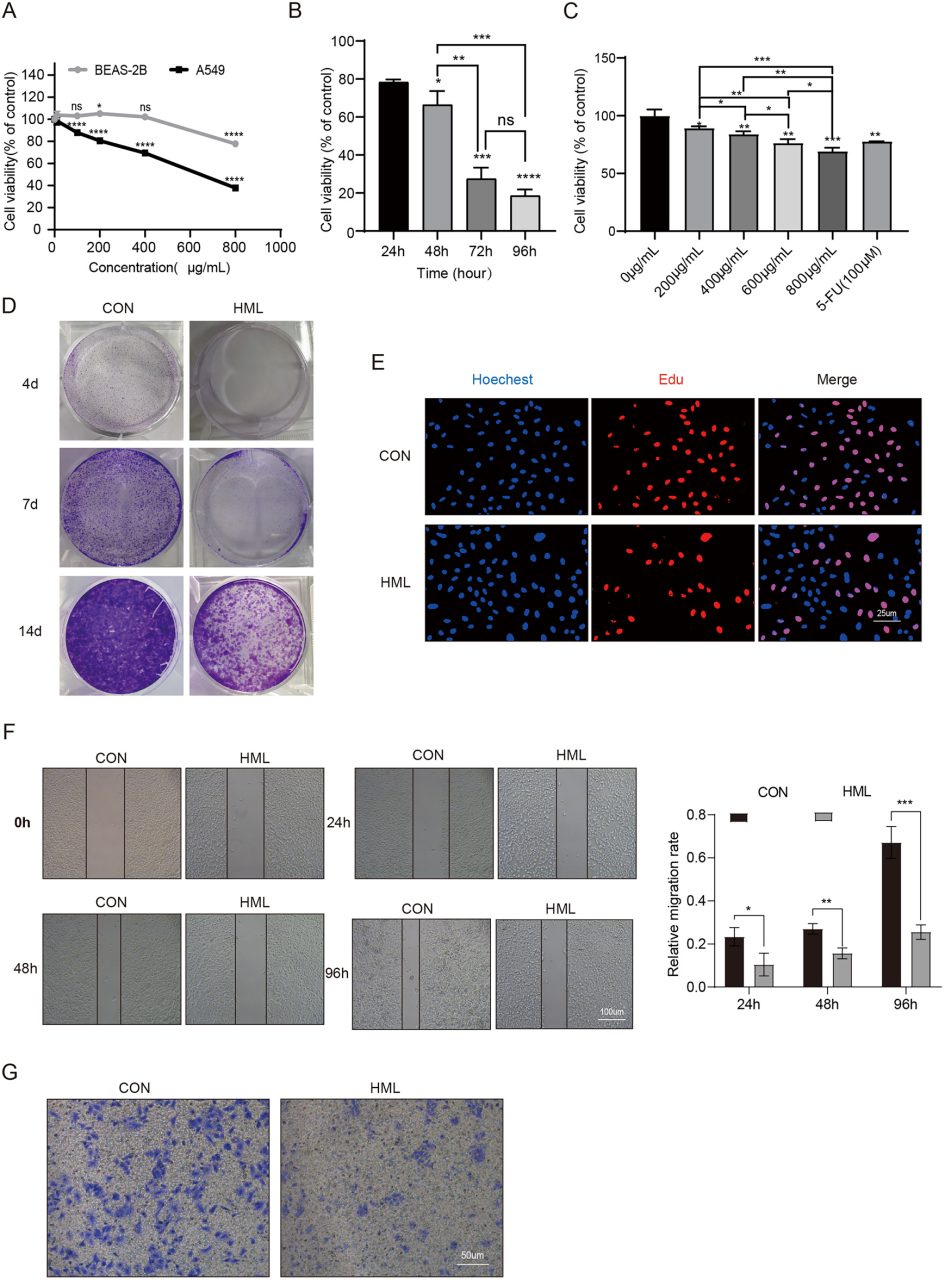
HML inhibited the proliferation, migration and invasion of A549 cells. (**A**–**C**) CCK-8 assays were performed to determine the cell viability, 5-FU: 100Um; (**D**) Representative image of colony formation experiments of the HML-treated experimental groups and the PBS-treated control groups. Each bar represents means ± SD from three independent; experiments; (**E**) The proliferation of A549 cell detected by EdU assay; (**F**) Scratch assay was performed on A549 cells after treatment with HML or PBS. The wound width was measured in 6 random sections, and the healing width was calculated by wound width at 0 h time point minus wound width at the measurement time point; (**G**) Transwell crystal violet staining method was used to detect cell migration and invasion ability. For (**B**–**E**) data are representative of 3 or more independent experimental replicates, and data are presented as mean ± SEM for all replicates. T-test: *P < 0.05, **P < 0.01, ***P < 0.001, ****P < 0.0001.

CCK8 assay demonstrated that when the concentration of HML was 400 μg/mL, it had no cytotoxicity to BEAS-2B cells, but significantly inhibited the growth of A549 cells (Fig. 1A). And HML exhibited this inhibition of A549 cells in a time- and dose-dependent manner (Fig. 1B,C). The clonogenic assays verified the long-term effects of HML on A549 cells, the number of cell colonies were decreased significantly after A549 cells were treated with HML at 400 μg/mL for 4, 7, and 14 days (Fig. 1D). EdU staining also showed that the proliferation of A549 cells was inhibited by HML (Fig. 1E). These results indicated that HML inhibited the proliferation of A549 cells even over a prolonged period.

Migration of tumor cells is a prerequisite of metastasis and invasion, as the translocation of tumor cells across extracellular barriers is a key step in metastasis. To determine the effect of HML on the migration of A549 cells, wound healing and transmigration assays were performed. The rate of migration of A549 cells treated with HML at 400 μg/mL was markedly decreased 10%, 16% and 26% at 24, 48 and 96 h compared with those in the control group respectively (Fig. 1F). The transwell assays indicated that the invasion of A549 cells decreased significantly compared with that of the control group in a time-dependent manner. (Fig. 1G). These findings suggested that HML may inhibit the transmigration and invasion of A549 cells.

### HML disturbed the cell cycle of A549

**Figure 2:**
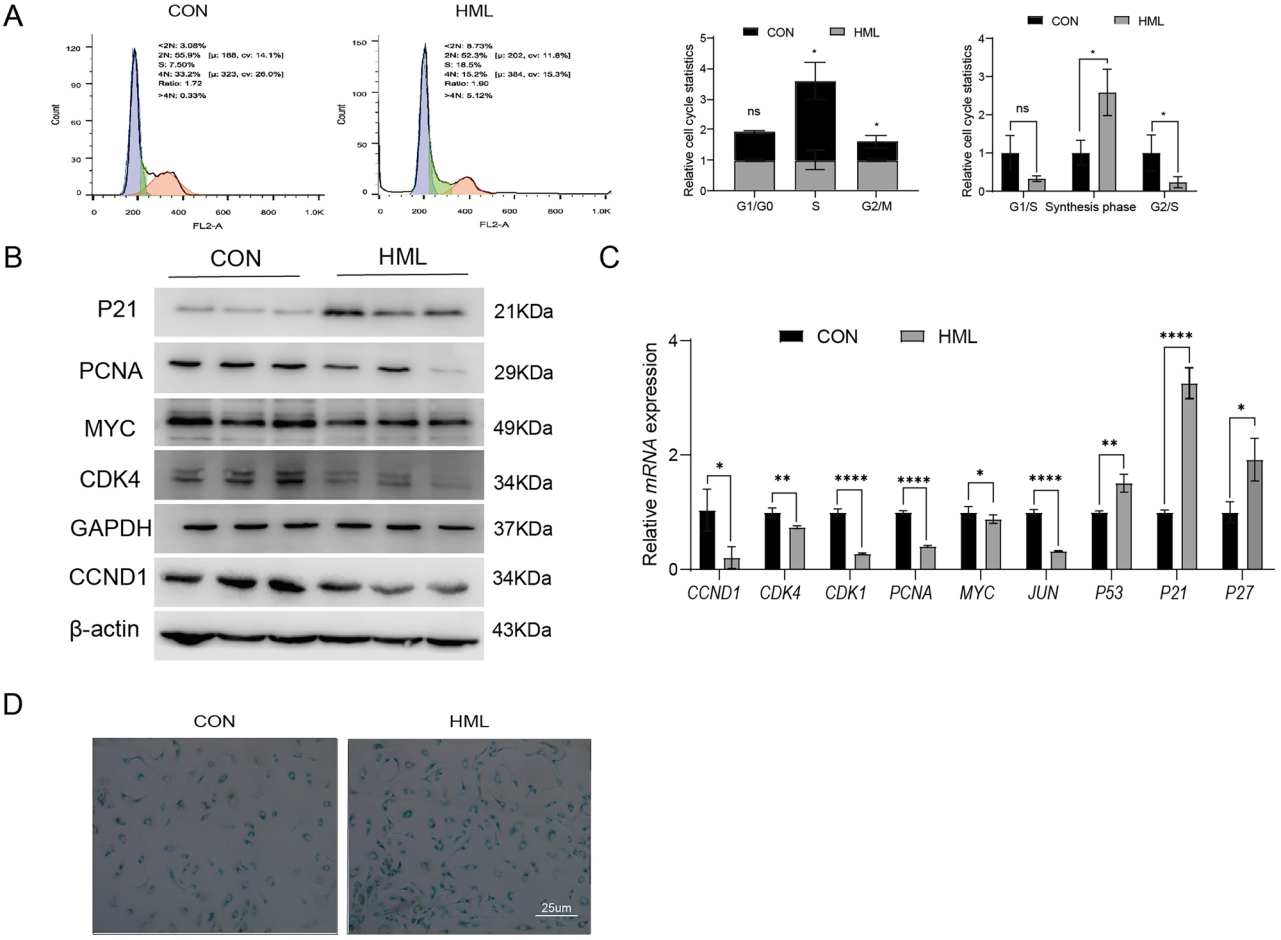
HML induced the cell cycle arrest and cell senescence. (**A**) Cell cycle distribution of HML against A549 was studied by flow cytometry. Profiles were obtained by FACS. The histogram shows the percentages of treatment groups relative to the normal control group at different stages of the cell cycle (CON = 1); (**B**) Western blot analysis was performed to confirm the alterations in CDK4, CCND1, P21 and MYC in total protein extracts from cells treated with HML or PBS (control). β-actin was used as a loading control. Representative results of three independent experiments are shown; (**C**) Quantitative real-time PCR (quantitative real-time PCR) determined the changes of cell cycle related genes *CCND1, CDK4, CDK1, PCNA, MYC, JUN, P53, P21* and *P27* at the transcriptional level; (**D**) SA-β-Gal staining was applied to detect cellular senescence following treatment with HML or control. The statistical significance of differences for 2 groups was determined by 2-sided *t* test and among 3 or more groups it was determined using 1-way ANOVA, followed by Sidak’s post hoc tests. *P < 0.05, **P < 0.01, ***P < 0.001, ****P < 0.0001.

To determine whether HML suppresses A549 cell proliferation by disrupting cell cycle progression, A549 cells were treated with 400 μg/mL HML for 48 h, and flow cytometry was used to analyze PI-stained cells. The results showed that administration of A549 cell with HML for 48 h resulted in a reduction in the G2/M phase populations and an increase in the S phase population (Fig. 2A). Western blotting and quantitative real-time PCR (RT‒qPCR) were used to measure the protein and mRNA expression levels of cell cycle-related genes in A549 cells after HML treatment. The protein levels of PCNA, MYC, CDK4, and CCND1 decreased significantly after HML treatment, while the protein expression of P21 increased dramatically (Fig. 2B). Consistent with the changes in the protein expression levels, the mRNA levels of CDK4, CDK1, and CCND1, DNA synthesis related genes of PCNA, MYC expression were downregulated, while P53, P21, and P27 were upregulated significantly (Fig. 2C). These findings collectively suggest that HML interrupted significantly cell cycle. Combined with the cell cycle analysis by flow cytometry, these data implied that complicated effect of HML on the cell cycle of A549.

To further validate whether the inhibition of cell proliferation was associated with the senescence, the acidic β-galactosidase activity was analyzed by senescence-associated β-galactosidase (SA-β-gal) staining after A549 cells were treated with HML for 48 h. As shown in Fig. 2D, HML led to a significant increase in the percentage of SA-β-gal-positive cells compared with the control. The results showed that HML promoted senescence of A549 cells.

### HML triggers mitochondrial damage

**Figure 3:**
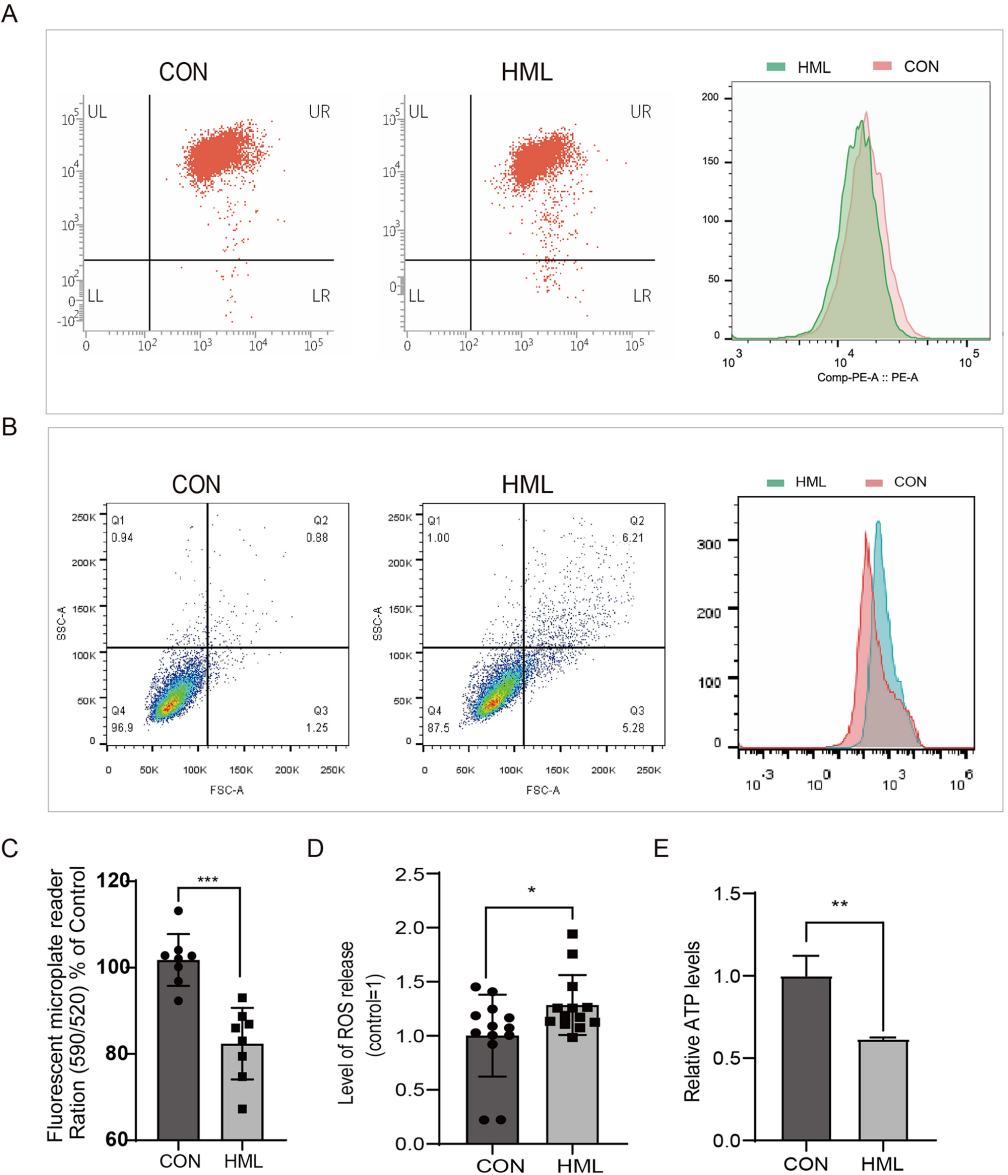
Effect of HML on mitochondria. (**A**,**B**) Flow cytometry analysis to determine changes in mitochondrial membrane potential (Δψm) (**A**) and ROS (**B**); (**C**,**E**) Fluorescence microplate reader analysis to determine changes in mitochondrial membrane potential (**C**) and ROS (**E**); (**D**) ATP level was determined by cell viability. *P < 0.05, **P < 0.01, ***P < 0.001.

Mitochondria have been proved to be damaged and even dysfunctional in many human cancers^13^. Mitochondrial membrane potential (MPP, Δψm) collapse is one of the signs of early cell apoptosis, and it is also an important indicator to detect mitochondrial function^14^. Excessive reactive oxygen species (ROS) is closely related to mitochondrial dysfunction and apoptosis^15,16^. Subsequently, the MMP, cellular ROS and ATP levels were evaluated after treatment with HML. The analyzing by flow cytometry and fluorescent probe staining revealed that MMP (Δψm) was decreased after HML treatment (Fig. 3A,C), the cellular ROS levels were elevated after HML treatment using flow cytometry and a fluorescence microplate reader (Fig. 3B,D), the intracellular ATP levels were significantly decreased as well (Fig. 3E). These observations suggest that HML induced the ROS production and reduction of mitochondrial membrane potential and ATP levels.

### HML inhibited ATG5- and ATG7-dependent autophagy through PINK1-mediated mitophagy in A549 cells

**Figure 4:**
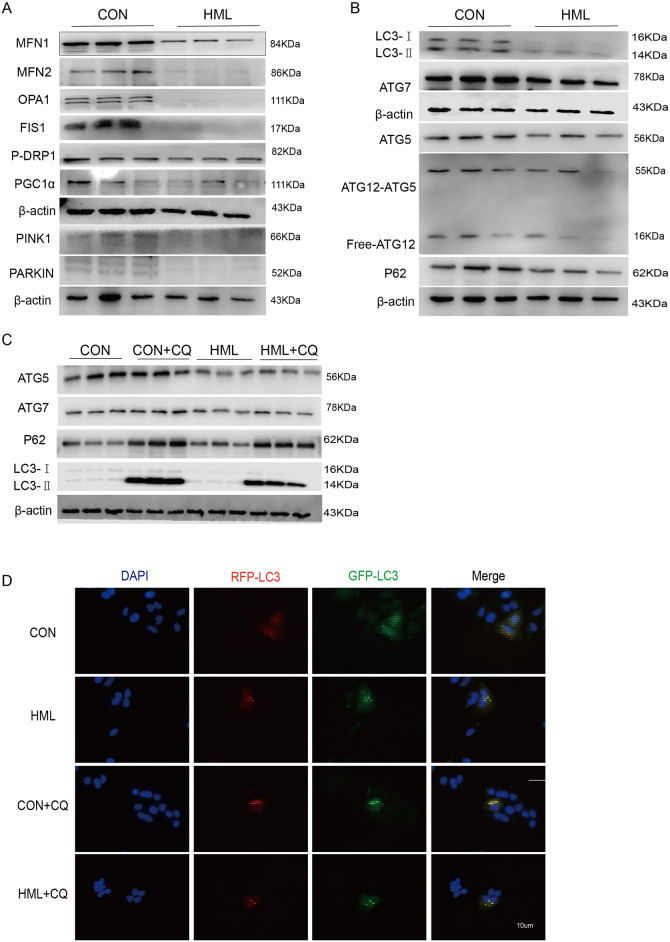
HML inhibits the initiation of autophagy. (**A**,**B**) Western blot analysis of mitochondrial and autophagy-related protein levels in A549 cells after 48 h of HML treatment; (**C**) Expression of ATG5, ATG7 and LC3II was detected in A549 cells by western blot analysis after inhibition of autophagic degradation with the autophagy inhibitor chloroquine. (**D**) GFP-LC3 dual fluorescent labeling indicator system analyzes changes in autophagic flux.

Given the decrease in MMP and the increase in intracellular ROS levels, we next examined whether HML induced the autophagy and mitophagy of A549. The expression levels of mitochondrial dynamics proteins (MFN1, MFN2, OPA1, FIS and p-DRP1), autophagy markers (LC3, ATGs and p62) and trigger mitophagy proteins (PINK1 and Parkin) were measured with the Western blotting (Fig. 4A). After HML treatment, the expression of mitochondria fusion and fission proteins MFN1/2, OPA1, FIS and p-DRP1 declined significantly, which imply the HML play a vital role on the mitochondria biogenesis progress. Then, we investigated changes in autophagy by a GFP-LC3 dual fluorescent labeling indicator system to monitor autophagic flux, we observed the decreased levels of PINK1 and Parkin caused by HML, which may trigger mitophagy in A549 cells. The autophagy-associated proteins LC3-II and LC3-I were notably decreased after HML treatment for 48 h, whereas the LC3-II/I ratio was appreciably increased, and the autophagy-related proteins P62, ATG7, ATG5 and ATG12 were downregulated compared to those in the control group (Fig. 4B). Autophagy was further inhibited after treatment with chloroquine, an inhibitor of LC3, for 6 h (Fig. 4C), we found that the autophagy flux was increased by HML, which determined by GFP-LC3 double fluorescent labeling indicator system (Fig. 4D). These findings demonstrated that HML impaired autophagy in A549 cells by inhibiting PINK1-mediated mitophagy.

### HML induced apoptosis in A549 cells though activation of the PTEN-p53 pathway

**Figure 5:**
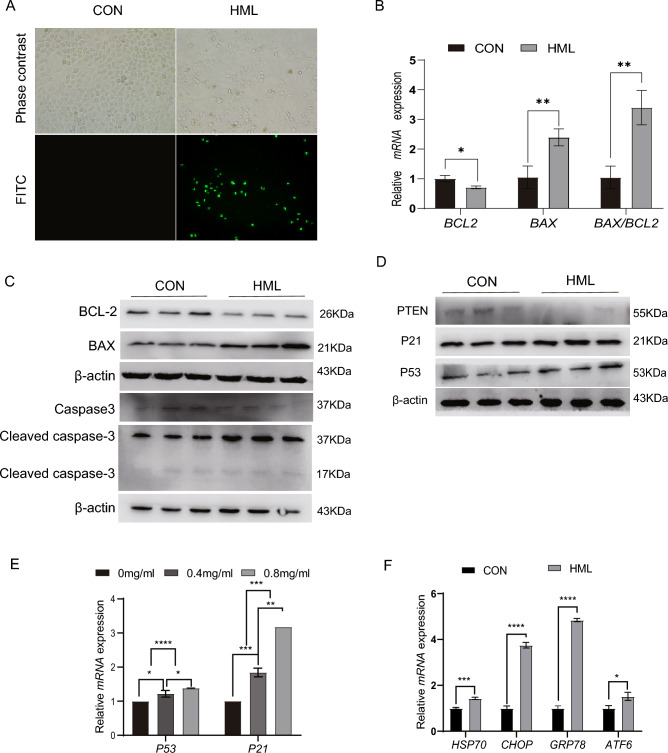
HML activates mitochondrial apoptosis pathway through PTEN/P53 signaling pathway. (**A**) Apoptosis rate of A549 cells were determined by TUNEL staining after HML treatment for 48 h; (**B**,**C**) RT-qPCR (**B**) and Western blot (**C**) detected changes in BAX and BCL2 expression levels; (**C**) Immunoblot analysis was performed to confirm the alterations in BAX, BCL2, P53, Cleaved Caspase-3 and Caspase-3. (**D**) Expression of HML and PTEN-P53-related pathway proteins (PTEN, P53, P21) were determined by western blot analysis at 0, 0.4, 0.8 μg/mL. (**E**) RT- qPCR analysis was performed on the translational and transcriptional levels of P53, and P21 in A549 cells after 48 h of HML treatment at 0, 0.4, 0.8 μg/mL. (**F**) RT-qPCR was performed on the translational and transcriptional levels of HSP70, CHOP, GRP78 and ATF6 in A549 cells after 48 h of HML treatment at the indicated concentrations. Data are presented as mean ± SEM. *P < 0.05, **P < 0.01, ***P < 0.001.

Apoptosis and cell cycle are intimately coupled. Some cell cycle checkpoints have a hand in cell division and programmed cell death^17^. Furthermore, autophagy plays an essential role in cellular homeostasis, but it can also become a cell death pathway when apoptosis is defective and under extreme stress conditions^18^. We next aimed to validate whether HML induces A549 cell apoptosis. TUNEL staining revealed that the ratio of apoptotic cells was increased after treatment with HML (Fig. 5A), the BAX expression level was up-regulated while the BCL-2 level was down-regulated in A549 cells after treatment with HML (Fig. 5B,C). An increased BAX/BCL-2 ratio contributes to apoptosis of A549 cells. Caspase-3 expression was significantly down-regulated while Cleaved Caspase-3 expression was up-regulated after treatment with HML. We measured the expression of P21, P53 and PTEN after the treatment of HML, P21, P53 were up-regulated whereas PTEN expression was down-regulated, as determined by Western blot analysis (Fig. 5D), the RNA level of P21, P53 were up-regulated in a dose-dependent manner (Fig. 5E). In addition, we found that endoplasmic reticulum (ER) stress-related genes HSP70, CHOP, GRP78 and ATF6 were significantly up-regulated at the mRNA level after HML treatment (Fig. 5F). These findings suggest that HML induced A549 cell apoptosis, which may be through PTEN/P53 pathway or/and by activating endoplasmic reticulum stress.

### HML synergistically enhances the therapeutic effects of 5-FU in A549 cells

**Figure 6:**
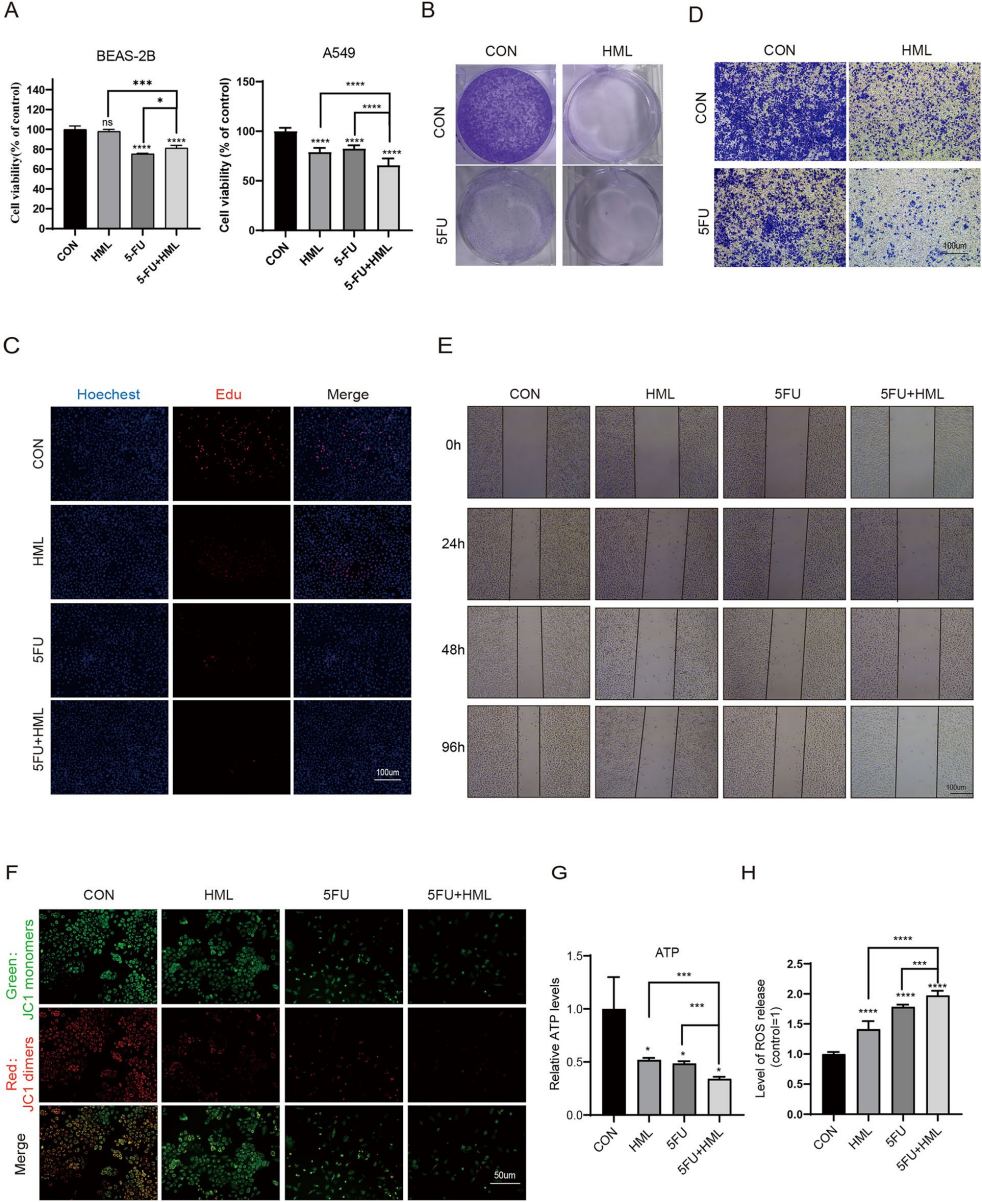
HML synergistically enhances the therapeutic effects of 5-FU in A549 cells. (**A**) CCK-8 assays were performed to determine the cell viability, 5-FU: 100uM; (**B**) Representative images of colony-forming experiments for different treatment groups. Each bar represents the mean SD of three independent experiments; (**C**) The proliferation of A549 cell detected by EdU assay; (**D**) Transwell crystal violet staining method was used to detect cell migration and invasion ability; (**E**) Scratch assay was performed on A549 cells after treatment with different processing. The wound width was measured in 6 random sections, and the healing width was calculated by wound width at 0 h time point minus wound width at the measurement time point; (**F**) Fluorescence diagram of typical JC1 staining. Red: JC1 dimers, Green: JC1 monomers; (**G**): ATP level was determined by cell viability; (**H**) Fluorescence microplate reader analysis to determine changes in ROS.

5-FU, a commonly used drug for the treatment of lung cancer, but is associated with systemic toxicity^19^. We next evaluated whether HML enhanced the effect of 5-FU in the lung cancer cells. As shown in Figs. 6A and 1A, CCK-8 assays showed that the combination of 5-FU and HML inhibited the growth of A549 cells by 66.84%, while the 5-FU or HML inhibited the proliferation of A549 cells by 80.29% or 78.57% respectively. Interestingly, the combination of HML and 5-FU increased the BEAS-2B cell viability from 75 into 81% compare to treatment with 5-FU. The same effect was confirmed by the colony-forming assay (Fig. 6B). Moreover, the A549 cell proliferative activity was significantly decreased in the combination treatment group in comparison to the control (Fig. 6C). As shown in the Fig. 6D and E, the combined treatment inhibited A549 cell migration and invasion obviously than 5-FU or HML alone. Finally, we found that the combined treatment group caused the decline of MMP and ATP levels, increased ROS level compared to HML treatment, 5-FU treatment group alone (Fig. 6F–H). Briefly, HML synergistically enhances the therapeutic effects of 5-FU.

## Discussion

Emerging body of evidence demonstrate natural products have been increasingly used in tumor prevention and treatment due to the advantages of safety^20^. Matrine extracts can inhibit the expression of MMP9 and NF-κB to inhibit the invasion of liver cancer cells^21^. Astragalus polysaccharide can alleviate the migration and invasion of cervical cancer, liver cancer and lung cancer^22,23^. In this study, we demonstrated the beneficial effects of HML against lung cancer cell which inhibits the growth and proliferation of A549 cells. HML induced apoptosis by activation of the PTEN-P53 pathway and inhibition of ATG5/7-dependent autophagy induced by PINK1-mediated mitophagy in A549 cells.

Cell cycle checkpoints are aberrantly expressed in various human cancers, and the CYCLIN-CDK-CKI signaling regulatory network ensures the precise control of the entire cell cycle^24,25^. Erik’s report illustrate the complex nature of cancer cell cycles, genes (CDK4, CDK6, CCND1, CDK2, and CCNE1) that regulate the G1/S transition yield particularly variant vulnerabilities in different cancer cell^26^. CYCLIN positively regulates CDK, while CKI negatively regulates CDK. CCND1 activates CDK4, which promotes cell entry into S phase together with CYCLIN E. In contrast, the KIP/CIP proteins P21 and P16 act as negative regulators that inhibit CYCLIN-CDK complexes^27^. PCNA and MYC are the major genes involved in the DNA synthesis. Our results showed that the downregulation of CDK4, CDK1, CCND1, PCNA and MYC, while the upregulation of P53, P21 and P27 in A549 cells after HML treatment. The cell cycle analysis exhibited that HML induced a reduction in the G2/M phase populations and an increase in the S phase population of A549. These findings indicated that HML involved in the G1/S transition, DNA synthesis in S phase, and G2/M phase. Autophagy is an evolutionarily conserved pathway whereby intracellular macromolecules such as cytoplasm and organelles are degraded in lysosomes into their constituent parts for recycling^28,29^. Mitophagy is a specific kind of autophagy^30^. LC3, a recognized autophagy marker, is mainly involved in the formation of autophagosomes and participates in the entire autophagy process^31^^,^^32^. The lysosomal turnover of the autophagosome marker LC3-II from cytosolic LC3-I reflects autophagic activity, and the amount of LC3-II is closely related to the number of autophagosomes. Furthermore, LC3 induces mitophagy to provide energy to cells and reduce ROS production. In the present study, we found that HML regulated the expression of mitochondrial dynamics proteins, including the mitochondrial fission and fusion proteins. Notably, the mammalian mitophagy pathway involving PINK1 and the E3 ubiquitin ligase Parkin were also decreased after HML treatment. However, despite the increased autophagic flux, autophagic initiation was inhibited. This compelling evidence suggests that HML inhibits ATG5- and ATG7-dependent autophagy, and LC3 is involved in autophagy induced by PINK1-mediated mitophagy in A549 cells.

Apoptosis occurs in a complex signaling cascade that is tightly regulated by multiple nodes, and loss of membrane asymmetry is a biochemical hallmark of apoptosis. We first found that the eversion of phosphatidylserine was increased and the Δψm was decreased in A549 cells, which indicated that HML induced apoptosis. Following the eversion of the cell membrane, the cell enters the process of programmed death. There are two main pathways for initiating cell death (intrinsic and extrinsic), both of which are initiated by Caspases^28,29^. As expected, Caspase-3 was downregulated, whereas Cleaved Caspase-3 was upregulated, further demonstrating the pivotal role of the mitochondrial apoptosis pathway in HML-induced A549 cell apoptosis. Overall, HML induces A549 cell apoptosis by activating caspase cascades and the mitochondrial apoptosis pathway.

The PTEN/P53 signaling pathway is considered a survival pathway due to its important role in tumor cell differentiation, proliferation, and survival^33^. P53, as a tumor suppressor protein, induces apoptosis by increasing the transcriptional activity of proapoptotic genes or suppressing the activity of antiapoptotic genes^34^, PTEN and P53 play critical roles in the DNA damage response and regulate the cell cycle and apoptosis^35,36^. Furthermore, ROS can cause DNA damage and then directly activate P53 and initiate apoptosis. Interestingly, the activation of mitophagy is also inseparable from the induction of PTEN^37,38^. In the current study, HML significantly suppressed the expression level of PTEN in A549 cells, whereas P53 was upregulated when PTEN was reduced, suggesting its potential role in HML-induced apoptosis in A549 cells.

ER stress is a typical response of cells to external injury. ER stress can resist injury by initiating the unfolded protein response (UPR). However, the persistent high level of ER stress can induce cells to enter the apoptosis pathway^39^. That is, sustained increases in ATF6 promote the isolation and translocation of 90-kDa ATF6 (P90) from GRP78/BiP to the Golgi apparatus and cleavage by SITE1 protease (S1P) in its luminal domain, releasing the 50-kDa cytoplasmic domain (P50)^40^. However, P50 (ATF6) enters the nucleus and initiates endoplasmic reticulum stress, inducing the increase of CHOP and GRP78. The increased CHOP could induce the increase of BAX and Caspase-3 and inhibit the expression of BCL2^41^. Our results showed that CHOP, GRP78, and ATF6 were all significantly increased, so we considered that HML might simultaneously activate ER stress and induce apoptosis.

**Figure 7:**
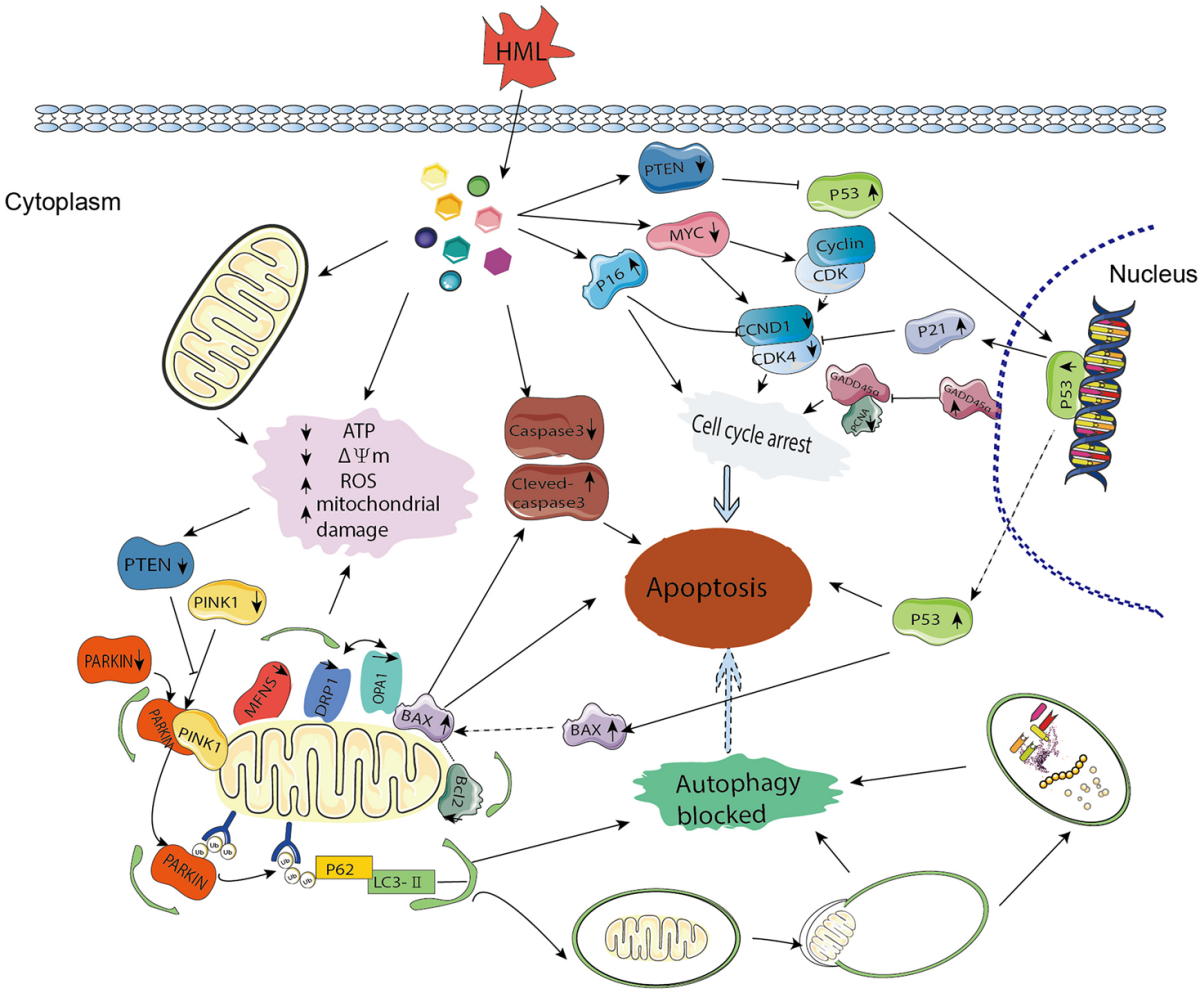
Graphical model of HML regulation of cell death in A549 cells.

In summary, HML inhibited A549 cell proliferation and autophagy, which induced apoptosis by activating the PTEN/P53 pathway (Fig. 7). In addition, our findings demonstrated the prospective capacity of HML with 5-FU through synergistic effect. Taken together, HML may serve as a potential tumor prevention and adjuvant treatment for its functional attributes.

## Data Availability

Data supporting the findings of this study may be contacted with the correspondent author.

## Abbreviations

HML: *Hibiscus manihot* L.
CCK-8: Cell counting kit-8
ROS: Reactive oxygen species
MMP: Mitochondrial membrane potential
ERS: Endoplasmic reticulum stress
RT-qPCR: Reverse transcription-quantitative polymerase chain reaction
DMEM: Dulbecco’s modified eagle’s medium
FBS: Fetal bovine serum
5-FU: 5-Fluorouracil
DAPI: 4,6-diamidina-2-phenylin
RIPA: Radio immunoprecipitation assay
SDS-PAGE: Sodium dodecyl sulfate-polyacrylamide gel electrophoresis
PI: Propidium iodide
BCA: Bicinchoninic acid assay
UPR: Unfolded protein response
TUNEL: Terminal deoxynucleotidyl transferase dUTP nick end-labeling
